# Human Astrocytes Model Derived from Induced Pluripotent Stem Cells

**DOI:** 10.3390/cells9122680

**Published:** 2020-12-13

**Authors:** Nicolas Leventoux, Satoru Morimoto, Kent Imaizumi, Yuta Sato, Shinichi Takahashi, Kyoko Mashima, Mitsuru Ishikawa, Iki Sonn, Takahiro Kondo, Hirotaka Watanabe, Hideyuki Okano

**Affiliations:** 1 Department of Physiology, Keio University School of Medicine, Tokyo 160-8582, Japan; n.leventoux@gmail.com (N.L.); s.morimoto.tmgh@gmail.com (S.M.); kent.imaizu@a5.keio.jp (K.I.); takashin@tka.att.ne.jp (S.T.); kymashima@gmail.com (K.M.); ishimi@keio.jp (M.I.); sonniki2017@gmail.com (I.S.); tkondo@keio.jp (T.K.); hwatanabe@keio.jp (H.W.); 2 Keio University Graduate School of Science and Technology, Kanagawa 223-8522, Japan; y.sato@brain.bio.keio.ac.jp; 3 Laboratory for Marmoset Neural Architecture, RIKEN Center for Brain Science, Wako City, Saitama 351-0198, Japan; 4 Department of Neurology and Stroke, Saitama Medical University International Medical Center, 1397-1 Yamane, Hidaka-shi, Saitama 350-1298, Japan

**Keywords:** astrocytes, iPSC, cell culture, disease modeling, neurodegenerative diseases

## Abstract

Induced pluripotent stem cell (iPSC)-based disease modeling has a great potential for uncovering the mechanisms of pathogenesis, especially in the case of neurodegenerative diseases where disease-susceptible cells can usually not be obtained from patients. So far, the iPSC-based modeling of neurodegenerative diseases has mainly focused on neurons because the protocols for generating astrocytes from iPSCs have not been fully established. The growing evidence of astrocytes’ contribution to neurodegenerative diseases has underscored the lack of iPSC-derived astrocyte models. In the present study, we established a protocol to efficiently generate iPSC-derived astrocytes (iPasts), which were further characterized by RNA and protein expression profiles as well as functional assays. iPasts exhibited calcium dynamics and glutamate uptake activity comparable to human primary astrocytes. Moreover, when co-cultured with neurons, iPasts enhanced neuronal synaptic maturation. Our protocol can be used for modeling astrocyte-related disease phenotypes in vitro and further exploring the contribution of astrocytes to neurodegenerative diseases.

## 1. Introduction

Accumulating evidence is now supporting the role of astrocytes in the initiation and maintenance of neurodegenerative diseases [1,2,3,4]. However, the exact contribution of astrocytes, the largest cell population in the central nervous system (CNS), to the development of neurodegenerative diseases has not been clarified due to limited accessibility to patients’ astrocytes. Human astrocytes and those of animal models exhibit essential differences in critical parameters such as the ratio of astrocytes to neurons [5], the spatial distribution and the complexity of astrocytes [6] or the molecular signatures and dominant signaling pathways [7]. The use of induced pluripotent stem cells (iPSCs) offers the possibility to circumvent these limitations by generating astrocytes of human origin in vitro. Previous studies have in fact demonstrated the interest of iPSC technology for studying the involvement of astrocytes in neurodegenerative diseases [8,9]. Unfortunately, the iPSC-derived astrocyte-like cells used in these studies were only partially characterized. Accurate modeling of astrocyte pathogenesis requires that iPSC-derived astrocytes recapitulate not only an astrocytic gene expression pattern but also perform the functions of genuine astrocytes. For example, it is necessary that the function of astrocytes in CNS energetic metabolism, through glycolysis, be investigated [10]. Astrocytes also regulate neuronal synapses by promoting synaptogenesis [11] or by modulating synaptic transmission through the transport of various amino acids, such as glutamate [12,13]. While previous reports have relied mainly on transcriptome and protein expression analyses [14] or calcium imaging studies [15,16] to characterize iPSC-astrocytes derived lines, only a few groups have performed co-culture with neurons [17,18] or investigated their glucose metabolism [19,20] and to the best of our knowledge, none has conducted all these analyses concurrently.

In the present report, we report an original protocol for generating iPSC-derived astrocytes (iPasts) and perform its validation at the cellular and functional levels. The gene expression profile of iPasts was compatible to human primary astrocytes and their functionality was confirmed in various aspects, including calcium dynamics, glutamate uptake, neuronal synapse maturation and glucose metabolism. Therefore, our protocol can generate iPasts that harbor key physiological features of human primary astrocytes. Considering the ever-increasing social and economic burdens of neurodegenerative diseases in our aging societies, iPasts represent an important new tool that will help to decipher the astrocyte-related mechanisms of neurodegeneration.

## 2. Materials and Methods

### 2.1. Research Involving Human Subjects

The study was conducted in accordance with the Declaration of Helsinki and the protocol was approved by the Ethics Committee of Keio University (No. 20080016, date of approval: 23 June 2020).

### 2.2. iPSC Culture

Human iPSCs were cultured on mitomycin-C-treated SNL murine fibroblast feeder cells in gelatin-coated (0.1%) tissue culture dishes. Human iPSCs were maintained in human ES medium (DMEM/F12, Wako, Tokyo) containing 20% KnockOut™ serum replacement (KSR, Gibco, Tokyo, Japan), 1% L-Glutamine 200 mM (Gibco), 0.8% non-essential amino acids (NEAA, Nacalai, Kyoto, Japan), 0.1 mM 2-mercaptoethanol (Sigma, St. Louis, MO, USA), 4 ng/mL fibroblast growth factor 2 (FGF-2, PeproTech, Tokyo, Japan) and Penicillin/Streptomycin at 37 °C in a humidified atmosphere of 3% CO_2_.

### 2.3. iPSC-Derived Astrocytes (iPasts) Induction In Vitro

**Embryoid Bodies (EBs) Formation Phase** (day 0 to 16): to differentiate iPSCs into iPasts, iPSC colonies were detached from feeder layers using a dissociation solution (0.25% trypsin, 100 μg/mL collagenase IV (Invitrogen, Carlsbad, CA, USA), 1 mM CaCl_2_ and 20% KSR) and cultured one night in suspension in ES medium without FGF-2 and with 10 µM Y-27632 (Nacalai, San Diego, CA, USA) to form EBs in a humidified atmosphere of 3% CO_2_. From day 1 to day 3, 3 µM dorsomorphin (Sigma), 3 µM SB431542 (Sigma) and 3 µM CHIR99021 (Focus Biomolecules, Philadelphia, PA, USA) were added to EB medium (DMEM/F12 containing 5% KSR, 1% L-Glutamine 0.8% NEAA, 0.1 mM 2-mercaptoethanol and Penicillin/Streptomycin) to enhance the differentiation into neural lineage. At day 4, the medium was changed to fresh EB medium supplemented with 1 µM retinoic acid (Sigma). At day 7, 10 and 13, the medium was changed to fresh EB medium with 1 µM retinoic acid and 1 μM purmorphamine (Cayman, Tokyo, Japan) and was maintained until day 16.

**Primary Neurosphere Formation Phase** (day 17 to 31): at day 16, EBs were enzymatically dissociated into single cells using TrypLE Select (Thermo Fisher Scientific, Waltham, MA, USA) and dissociated cells were cultured in suspension at a density of 1 × 10^5^–4 × 10^5^ cells/mL in neurosphere medium consisting of media hormone mix (MHM) [21] supplemented with 2% B-27 (Nacalai), 0.8% NEAA, 1 μM purmorphamine, 20 ng/mL FGF-2, 10 ng/mL epidermal growth factor (EGF, PeproTech, Rocky Hill, NJ, USA) in a humidified atmosphere of 5% CO_2_. The medium was changed every 4–6 days to form primary neurospheres.

**Secondary Neurospheres Formation Phase** (day 32 to 47): at day 32, primary neurospheres were dissociated as described above and cultured at a density of 1 × 10^5^–4 × 10^5^ cells/mL in neurosphere medium without purmorphamine.

**Terminal Differentiation Phase** (A0 iPast: day 48 to 76): at day 48, secondary neurospheres were dissociated and cultured at a density of 4 × 10^5^–1 × 10^6^ cells/well in 6-well plates coated with 200× diluted growth-factor-reduced Matrigel (Corning, Corning, NY, USA) and iPasts differentiation medium consisting of MHM supplemented with 2% B-27, 0.8% NEAA, 10 ng/mL of brain-derived neurotrophic factor (BDNF, R&D, Minneapolis, MN, USA), 10 ng/mL of glial cell-derived neurotrophic factor (GDNF, Alamone, Tel Aviv, Israel) in a humidified atmosphere of 5% CO_2_. The medium was changed every 4–7 days depending on the medium color.

**Astrocytes Purification Phase.** A1 iPasts (day 77 to 83): at day 77, A0 iPasts were enzymatically dissociated into single cells using Accutase (Nacalai) and the dissociated cells were plated at a density of approximately 1 × 10^6^ cells/well in 6-well plates coated with 200× diluted growth-factor-reduced Matrigel and cultured in iPast-differentiation medium. The medium was changed after 3–4 days depending on the medium color. A2 iPasts (day 84 to 90): A1 iPasts were dissociated and cultured as described above. A3 iPasts (day 91 to 97): A2 iPasts were dissociated and cultured as described above. A4 iPasts (from day 98): at day 98 iPasts were dissociated as described above and used for experiments. A4 iPasts were also stored in serum-free CellBanker2 (Zenoaq, Koriyama, Japan) at −196 °C.

### 2.4. RNA-Sequencing (RNA-Seq)

For RNA-Seq, RNA from EBs, neurospheres and iPasts were extracted using RNeasy kit (QIAGEN, Hilden, Germany). The indexed cDNA libraries were prepared using the Nextera XT library preparation kit (Illumina, Tokyo, Japan) and were sequenced using a NovaSeq6000 (Illumina) to obtain 150-bp paired-end reads. Published RNA-Seq data of primary astrocytes [7] and iPSC-derived astrocytes [22,23] were downloaded via the National Center for Biotechnology Information (NCBI) Short Read Archive (Supplemental Table S1). Raw FASTQ files were trimmed for adapters by Cutadapt [24] and aligned to the GRCh37 genome build using HISAT2 [25]. Counts were calculated using featureCounts [26] and normalized by variance-stabilizing transformation using the DESeq2 [27]. Differentially expressed genes were identified using a cutoff of 0.01 for Benjamini-Hochberg adjusted *p*-values and a cutoff of 4 for fold-change ratio. A comparative analysis of our dataset with fetal and adult astrocytes was performed across a set of astrocyte-specific genes used in Tchieu et al. [23] (Supplemental Table S2). The RNA-Seq dataset including raw data and preprocessed data has been deposited in the NCBI Gene Expression Omnibus and is accessible through GEO series accession number GSE161024.

### 2.5. Quantitative RT-PCR

Total RNA was isolated with a RNeasy mini kit or micro kit (QIAGEN) with DNase I (QIAGEN) treatment and cDNA was prepared by using an iScript cDNA Synthesis Kit (Bio-Rad, Tokyo, Japan). Quantitative RT-PCR was performed using SYBR Premix Ex Taq II (Takara Bio, Kusatsu, Japan) on a ViiA 7 Real-Time PCR System (Thermo fisher Scientifics, Tokyo, Japan). The details of qRT-PCR primers are described in the Supplementary Table S3.

### 2.6. Control Human Astrocyte Lines

For quantitative RT-PCR, total RNA from human fetal astrocytes (ScienCell, #SCR 1815, Cosmobio, Tokyo, Japan) (referred to as FA in the text) and a frozen sample from the frontal lobe of a 75-year-old male (referred to as HB in the text) were used. The use of this human sample was approved by the committee of Ethics of Mihara Memorial Hospital (Approval No. 087-03).

For cell culture, human fetal astrocytes (referred to as FAC in the text) from Lonza Pharma&Biotech (#CC-2565), grown according to the manufacturers’ instructions, were used.2.7. Immunofluorescence Analysis.

Cells were fixed with 4% paraformaldehyde for 20 min at room temperature and then incubated with blocking buffer (PBS containing 2% normal goat serum, 2% BSA and 0.2% Triton X-100) for 1 h at room temperature. Cells were then incubated overnight at 4 °C with primary antibodies (Supplemental Table S3), diluted in blocking buffer without Triton X-100. The cells washed with PBS were then incubated with secondary antibodies Alexa Fluor 488, Alexa Fluor 555 or Alexa Fluor 647 (Supplemental Table S3) and 10 µg/mL Hoechst 33258 (Dojindo, Shanghai, China) for 1 h at room temperature. Slides/coverslips were mounted with Permafluor (Thermo Fisher).

### 2.7. Microscopy

Cells were examined with a BZ-9000 microscope (Keyence, Osaka, Japan), IN Cell Analyzer 6000 (Cytiva, Tokyo, Japan) and LSM700 (Zeiss, Oberkochen, Germany) confocal microscope. Bright field images were captured using Nikon DS-L3 system (Nikon, Tokyo, Japan).

### 2.8. Calcium Imaging

Astrocytes induced from iPSCs by culture in iPast-differentiation medium were cultured for 5 days before performing calcium imaging assay. Cells were washed once with PBS, then loaded with 1 μg/mL Fluo-8 AM (AAT Bioquest, Sunnyvale, CA, USA) in recording medium (20 mm HEPES, 115 mm NaCl, 5.4 mm KCl, 0.8 mm MgCl_2_, 1.8 mm CaCl_2_ and 13.8 mm glucose-Dojindo) containing 0.02% Cremophor EL (Dojindo) and incubated for 20 min at 37 °C and 5% CO_2_. After washing with PBS, the medium was changed for the recording medium. Fluorescent images were obtained using an IX83 inverted microscope (Olympus, Kyoto, Japan) equipped with an Electron Multiplying CCD Camera (Hamamatsu Photonics, Hamamatsu, Shizuoka, Japan) and LED illumination system pE-4000 (CoolLED, Andover, UK). MetaMorph Image Analysis Software (Molecular Devices, Tokyo, Japan) was used to analyze the live cell calcium traces. Six hundred and one frames were recorded at 2 Hz using the stream acquisition mode. For imaging data analysis, a miniscope 1-photon-based calcium imaging signal extraction pipeline (MIN1PIPE) software was used [28]. This software automatically subtracts the background and corrects plate moves occurring during acquisition. Locations of astrocytes and region of interests were then extracted. The rising phase of each calcium transient is identified as the calcium event when the 1st derivative of Δ*F*/*F*_0_ = $\frac{F\left(t\right)-F_{0}}{F_{0}}$ (where *F(t)* is the intensity of fluorescence at ‘*t*’ and *F*_0_ the intensity of fluorescence at the beginning of the rising phase) rises above 0 and continues to increase above 2 standard deviations of baseline fluctuation.

### 2.9. Glutamate Uptake Assay

iPasts were seeded at a density of 20,000 cells per well in a 24-well plate in iPast cell culture medium for seven days. On day 7, medium was removed and replaced by MHM containing glutamate at the concentration of 250 µM. Glutamate concentration after 1, 2 and 4 h was measured using the L-Glutamate assay kit (Yamasa Neo, Choshi, Japan) on an iMark microplate reader (Bio-Rad), according to the manufacturer’s protocol. Concentration of glutamate were normalized to the total protein concentration measured also on iMark microplate reader by BCA protein method (ThermoFisher).

### 2.10. Co-Culture of iPasts With Neurons

Cortical neuronal induction of iPSCs was performed as described previously [29,30] with some modifications. Briefly, adhesive iPSCs were cultured with dual SMAD inhibitors and a Wnt inhibitor to obtain forebrain neural precursors. These precursors were then cultured with GDNF, BDNF, ascorbic acid, dibutyryl cAMP and γ-secretase inhibitor, to obtain forebrain excitatory neurons. Three days after plating 2 × 10^5^ iPasts per 6.5 mm Matrigel-coated polycarbonate trans-well inserts (0.4 µm pores) (Corning), 1 × 10^5^ neurons derived from 1210B2-iPSCs were seeded and co-cultured with iPasts in Neurobasal medium (Gibco) supplemented with 2% B-27 for additional 5 days before immunofluorescent staining with anti-Synapsin-1 and anti-MAP2A antibodies (Supplemental Table S3). Cells were imaged using a LSM710 confocal microscope and Synapsin-1+ puncta on MAP2+ neurites were measured by IN Cell Analyzer 6000 (Cytiva).

### 2.11. Measurement of the Rate of D-[1-^14^C]-glucose and D-[6-^14^C]-glucose Oxidation to ^14^CO_2_

D-[1-^14^C]-glucose (2.035 GBq/mmol) and D-[6-^14^C]-glucose (2.035 GBq/mmol) were obtained from American Radiolabeled Chemicals. Two hundred thousand iPasts suspended in iPast-differentiation medium with 33 mM D-glucose were placed into 12.5 cm² culture flasks coated with 200× diluted growth-factor-reduced Matrigel at 37 °C in humidified air containing 5% CO_2_. The cells were used for assays when they had reached confluence (typically on day 4). The rate of [^14^C]-glucose oxidation to ^14^CO_2_ was measured as previously described with some modifications [31]. Briefly, cells were washed twice with glucose-free PBS; then 2 mM D-glucose labeled with 1 µL/mL D-[1-^14^C]-glucose or D-[6-^14^C]-glucose (original concentrations: 3.7 MBq/mL) in Dulbecco’s balanced salt solution (110 mM NaCl, 5.4 mM KCl, 1.8 mM CaCl_2_, 0.8 mM MgSO_4_, 0.9 mM NaH_2_PO_4_ and 44 mM NaHCO_3_) were added and cells were incubated at 37 °C for 60 min. Flasks were capped with rubber stoppers containing a center well (Kimble/Kontes) with a cotton ball containing 100 µL of hyamine hydroxide 10-X (PerkinElmer, Waltham, MA, USA), through which ^14^CO_2_ was trapped. The reactions were terminated by the injection of 250 µL of 60% perchloric acid through the rubber stopper and the flasks were kept at 4 °C overnight to trap ^14^CO_2_. The cotton balls were then transferred to 20-mL glass scintillation counter vials and 500 µL of ethanol and 10 mL of Insta-Fluor Plus (PerkinElmer) were added. The ^14^C content of the vials was evaluated using a liquid scintillation counter (Tri-Carb 3100TR; PerkinElmer Life Sciences). Because a substantial ^14^C count can be obtained from a flask without cells [32], the ^14^C count obtained from a cell-free flask in which the reaction had been stopped at 60 min was regarded as the background value. The cell layers remaining in flasks after the removal of the reaction mixtures were digested with 5 mL of 0.1 mol/L NaOH and their protein contents were determined. We measured the rates of total glucose oxidation (pmol glucose/µg protein/60 min) based on the conversion from [1-^14^C]-glucose to ^14^CO_2_ over 60 min. TCA cycle consumption was measured by the conversion rate from [6-^14^C]-glucose to ^14^CO_2._ Pentose phosphate pathway (PPP) activity was calculated as the difference between the total glucose consumption and the TCA cycle consumption [33,34].

### 2.12. Statistical Analyses

The statistical tests and sample sizes (n) are indicated in the respective figure legends. Tests were performed using GraphPad software (v8.2.1). Significances: ****, ***, **, * indicate *p*-values (p) < 0.0001, < 0.001, < 0.01 and ≤ 0.05, respectively.

## 3. Results

### 3.1. Differentiation of 201B7 and WD39 iPSC Lines Into iPasts

**Figure 1:**
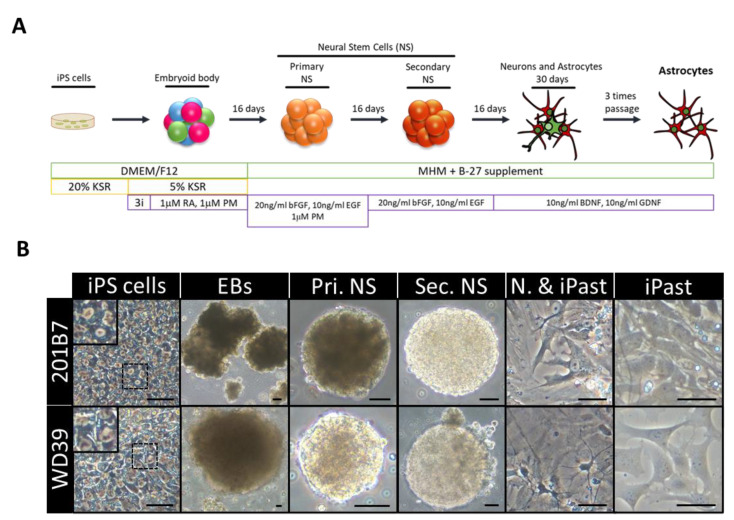
Induction of Human Astrocytes (iPasts). (**A**) Design of the human iPast induction protocol under serum free condition. Abbreviations: DMEM/F12: Dulbecco’s Modified Eagle Medium: Nutrient Mixture F-12; MHM: Media Hormone mix; B-27™: optimized serum-free supplement; KSR: KnockOut™ Serum Replacement; 3i: 3 µM CHIR99021, 3 µM 431542, 3 µM Dorsomorphine; RA: Retinoic acid; PM: Purmorphamine; bFGF (FGF-2): Basic fibroblast growth factor; EGF: Epidermal Growth Factor; BDNF: Brain-Derived Neurotrophic Factor; GDNF: Glial cell line-Derived Neurotrophic Factor. (**B**) Representative images of cells at each step of iPast induction process from the two iPSC control lines 201B7 and WD39. Abbreviations: EBs: Embryoid Bodies; Pri. NS: primary neurospheres; Sec. NS: secondary neurospheres; N. & iPasts: Neurons and iPasts; iPasts: human iPSC-derived astrocytes. The cells in the insets at iPSC stage are higher magnifications of cells in dashed boxes and indicative of good quality iPSCs with clear perinuclear halos. Scale bars: 50 µm (20 µm for iPast stage).

In the present study, we used as starting material two control iPSC lines that were previously reported—201B7 iPSC line derived from a 36-year-old healthy female [35] and WD39 iPSC line from a 16-year-old female [36]. We first evaluated the expression of the pluripotency markers SOX2, NANOG, SSEA4 and Tra1-81 by immunofluorescence to assess the quality of iPSCs (Figure S1A). Embryoid bodies (EBs) were generated from iPSCs and were directedly differentiated toward the neural lineage by the treatment with the bone morphogenetic protein inhibitor Dorsomorphin and the TGFβ inhibitor SB431542 to prevent the differentiation toward mesendodermal and non-neural ectodermal lineages [37] and with the GSK3β inhibitor CHIR99021, to direct the growth of embryoid cells into stable neuronal and glial differentiation [38] (Figure 1A). To commit the cells into the neural lineage, EBs were grown with retinoic acid [39] and purmorphamine [40] from day 7. Cells were then expanded for one month as neurospheres (NS) and differentiated into neural cells in adherent culture with brain-derived neurotrophic factor (BDNF) and glial cell-derived neurotrophic factor (GDNF) for three additional weeks. At this time point, neuron-like cells with neurites were observed, along with astrocyte-like cells with characteristic processes (Figure 1B). To eliminate neuron-like cells and purify astrocytes from the culture, the cells were repeatedly dissociated and replated. Neuron-like cells could not survive during the passages and their number decreased in culture from one passage to the other, resulting in the enrichment in astrocytic-like cells.

Finally, the obtained cells exhibited a homogenous astrocytic morphology, which prompted us to proceed to their cellular and functional characterization (Figure 1B).

### 3.2. iPasts are Transcriptionally Committed to the Astrocytic Lineage

**Figure 2:**
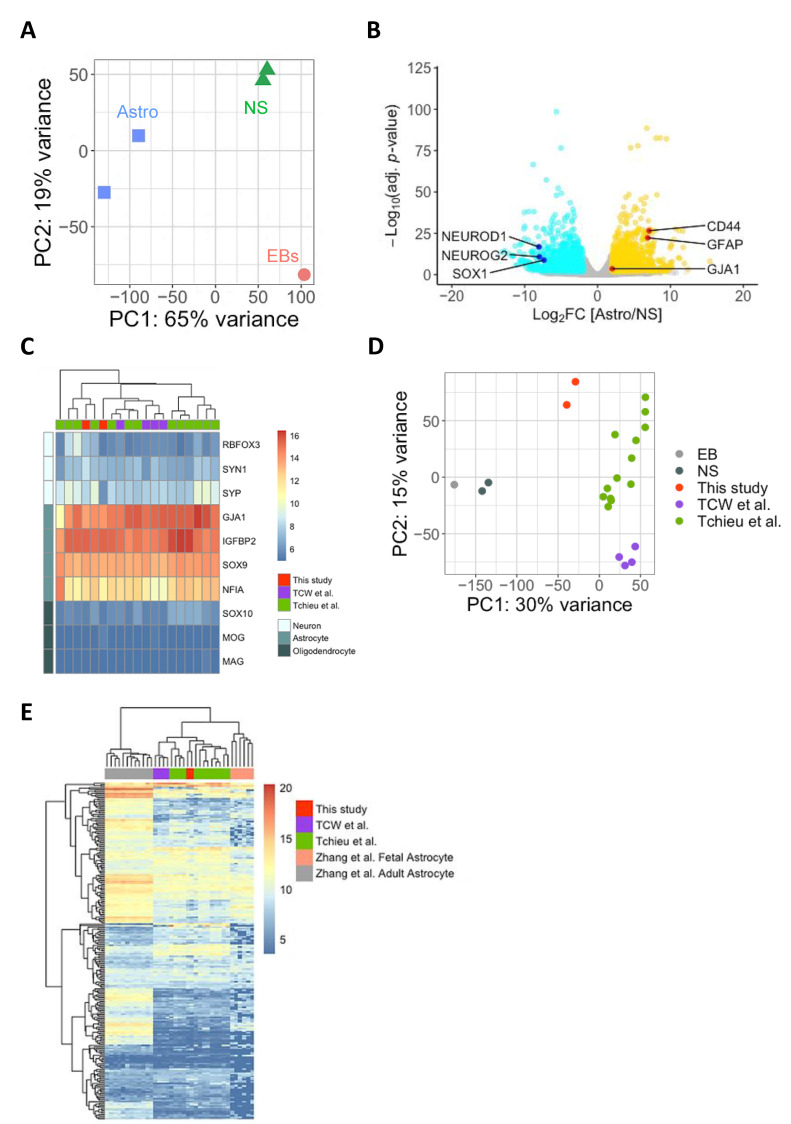
Transcriptome Analyses of iPasts. (**A**) Principal component analysis (PCA) plot of iPSC-derived cells differentiated along astrocyte lineage by the iPast protocol. (**B**) Volcano plot showing differential expressed genes between neurospheres (in blue) and astrocytes (in yellow). (**C**) Heatmap showing the expression of trilineage markers in iPSC-derived astrocytes obtained by the iPast protocol or previously described protocols. (**D**) PCA plot of iPSC-derived astrocytes, EBs and NS. (**E**) Heatmap of genes associated with astrocyte identity in iPSC-derived astrocytes and human primary astrocytes.

First, we examined by RNA sequencing (RNA-Seq) the transcriptomic changes during the process of cell differentiation from the state of embryoid bodies to iPasts. Principle component analysis (PCA) revealed that the gene expression profiles of iPasts were different from EBs and NS (Figure 2A): 1050 and 964 genes were significantly upregulated in iPasts and NS, respectively, compared to EBs. The neural stem cell marker *SOX1* [41] and master regulators of neuronal differentiation, *NEUROG2* and *NEUROD1* [42], were highly upregulated in NS. On the other hand, genes known to be expressed by astrocytes such as *CD44* [43], *GFAP* and *GJA1* [44], were enriched in iPasts (Figure 2B). We then carried out a meta-analysis to compare the results of the RNA-Seq of iPasts with those of iPSC-derived astrocytes obtained by previously reported protocols [22,23] (Supplementary Table S1). We examined the expression of gene markers related to the neuronal, oligodendrocytic and astrocytic lineages (Figure 2C). All iPSC-derived astrocytes, including iPasts, expressed high levels of astrocytic markers and, conversely, poorly expressed neuronal/oligodendrocytic markers. Next, we compared these iPSC-derived astrocytes by PCA and found that the two lines of iPasts appeared closer to each other than astrocyte-lines induced by other protocols were to their relatives (Figure 2D). Finally, we examined how close iPasts were from fetal and adult human astrocytes [7]. A comparative analysis of iPasts RNA-Seq data with those of fetal and adult human astrocytes revealed that iPasts were transcriptionally closer to fetal human astrocytes than to adult astrocytes (Figure 2E).

These results indicate that iPasts have a transcriptional profile similar to previously reported iPSC-derived astrocytes, which resembles primary fetal astrocytes.

### 3.3. iPasts are Transcriptionally Close to Fetal Astrocytes

**Figure 3:**
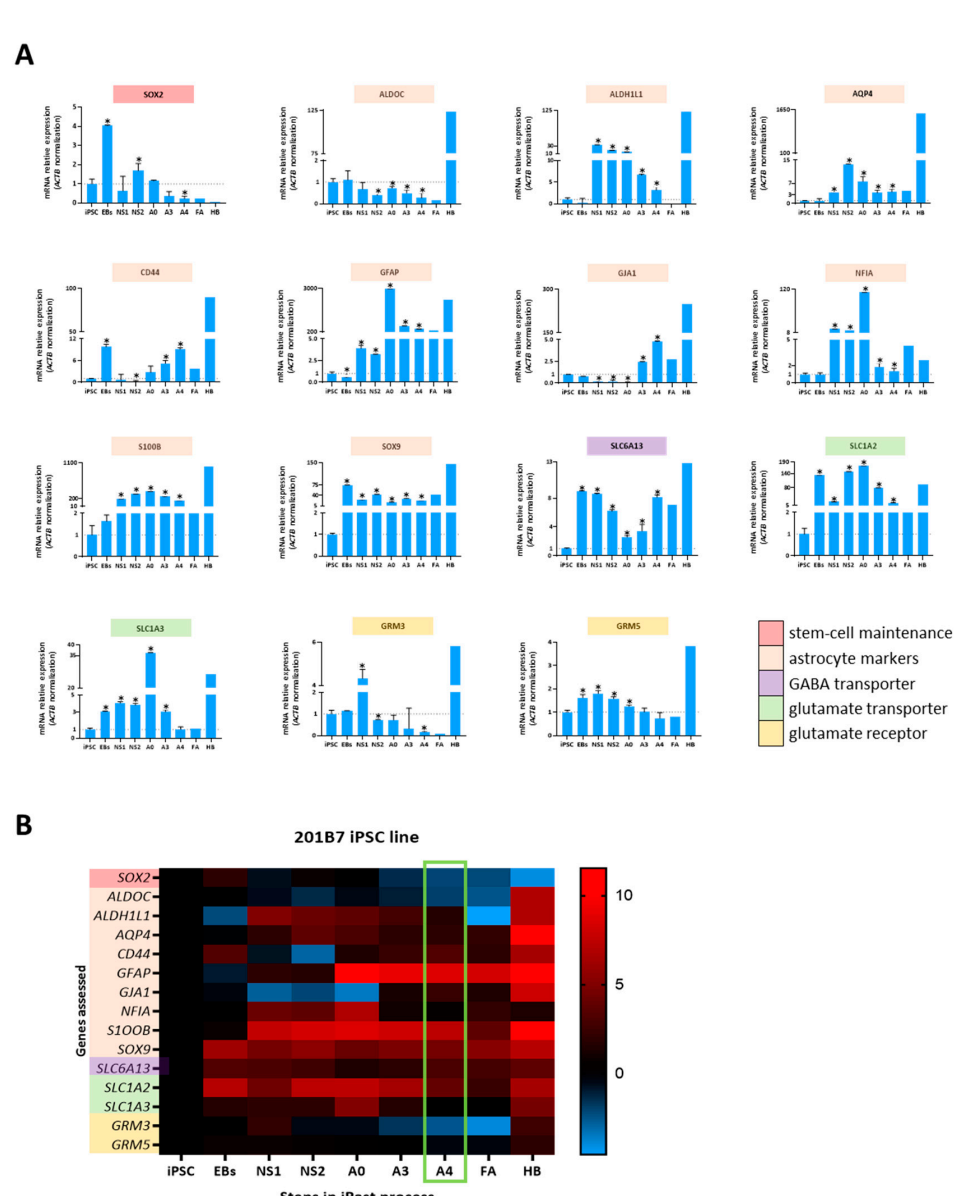
Longitudinal Analysis by Quantitative PCR During iPast Induction Protocol. (**A**) Quantitative real-time polymerase chain reaction of indicated markers in cells derived from the 201B7 iPSC line. Markers are arranged by family according to the indicated color code (stem-cell maintenance, astrocyte markers, GABA transporter, glutamate transporters and receptors). FA stands for control fetal astrocytes and HB for human brain. Values are normalized to *ACTB* quantification and represent the fold change ratio value relative to iPSC value. *n* = 3 passages in duplicate. Data shown are mean ± SEM. Statistical test used: Wilcoxon Matched-pairs test. * indicates *p*-value ≤ 0.05 (**B**) Heatmap summing up gene transcription by quantitative RT-PCR in A. iPasts A4 status is highlighted by the green box. Values are logarithm to base 2 of fold changes from A.

To support the transcriptomic data from RNA-Seq, quantitative real-time polymerase chain reaction (RT-PCR) analyses were performed (Figure 3A,B and Figure S2A,B). Genes involved in neural stem cell maintenance (*SOX2*), astrocyte differentiation (*ALDOC*, *ALDH1L1*, *AQP4*, *CD44*, *GFAP*, *GJA1*, *NFIA*, *S100B* and *SOX9*), GABA transport (*SLC6A13*), glutamate transport (*SLC1A2* and *SLC1A3*) and glutamate receptors (*GRM3* and *GRM5*) were selected. A fetal astrocyte cell line (referred to as FA) and a human brain sample (referred to as HB) were used as control samples. The expression of the transcription factor *SOX2*, which indicates an undifferentiated state in neural stem cells, was high in EBs and, to a lesser extent, in NS, whereas its expression was gradually decreased in iPasts along passaging. Conversely, most of the astrocyte differentiation markers were upregulated along iPasts differentiation. Importantly, the expression levels of those markers in iPasts were similar to their level in human FA. We also noted a statistically significant burst in the transcription of *SLC1A3,* observable in A0-step for the cell line 201B7 and in A3-step for the cell line WD39. For both iPast lines, the expression level of *SLC1A3* in A4-step had decreased to the level in FA. Consistently, *SLC1A2* was significantly increased in A4-iPasts compared to FA. These results demonstrate the mature differentiation of iPasts as *SLC1A3* is known to be preferentially expressed in the developing brain, whereas *SLC1A2* is found in the adult brain [45]. Conversely, *GRM3* and *GRM5*, known to be highly expressed in adult human brains, were only weakly expressed in iPasts and FA.

Collectively, our results indicate that A4-iPasts are transcriptionally closer to fetal astrocytes rather than to mature astrocytes.

### 3.4. iPasts Express Proteins of the Astrocytic Lineage

**Figure 4:**
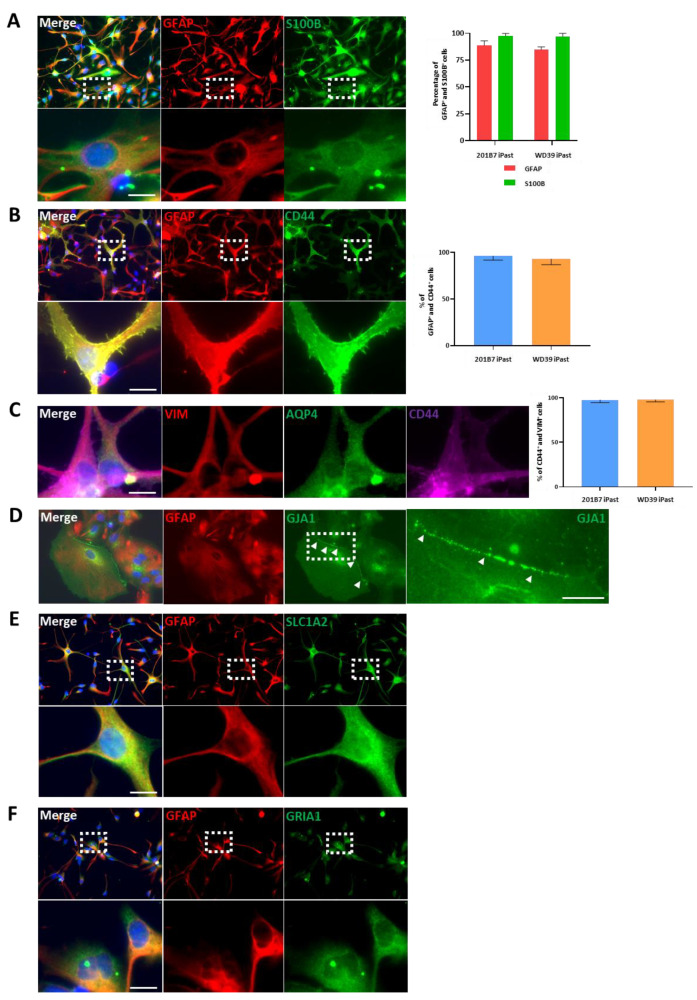
Representative Immunofluorescence Stainings of 201B7 iPasts. (**A**) GFAP (red) and S100B (green) immunostaining of 201B7 iPasts. Lower pictures are high magnification of white dotted-line boxes. Right-hand panel, quantitative analyses of GFAP+ and S100B+ cells from 5 fields (10,000 µm²) for 201B7 and WD39 iPasts. (**B**) GFAP (red) and CD44 (green) immunostaining. The lower panel is a high magnification of the white dotted-line boxes. Right-hand panel, percentage of GFAP+/CD44+ from 5 fields (10,000 µm²) for 201B7 iPasts and WD39 iPasts. (**C**) Immunostaining for Vimentin (VIM) (red), AQP4 (green) and CD44 (magenta). Right-hand panel, analysis of CD44+/VIM+ cells from 5 fields (10,000 µm²) for 201B7 iPasts and WD39 iPasts. (**D**) Immunostaining for GFAP (red) and GJA1 (green). Arrowheads point to GJA1+ gap junction proteins. The picture on the extreme right is a high magnification of the adjacent white dotted-line box. (**E**) Immunostaining for GFAP (red) and SLC1A2 (green). The lower panel represents a high magnification of the corresponding white dotted-line boxes. (**F**) Immunostaining for GFAP (red) and Glutamate Ionotropic Receptor AMPA Type Subunit 1 (GRIA1) (green). Bottom pictures are high magnifications of white dotted-line boxes. Nuclei are stained with Hoechst 33258. *n* = 3 independent astrocytes induction experiments. Scale bars: 10 µm.

After having characterized iPast lines at the RNA (transcriptome) level, we examined by immunofluorescence the expression of various proteins: TUBB3 (neuronal lineage), O4/OLIG2 (oligodendrocytic lineage) or AIF1 (microglia/macrophage activation marker) could not be detected (Figure S1B,C, upper panels) in iPasts. The effective detection of these proteins with our antibodies was confirmed using positive controls (Figure S3A–C,E). We then sought to determine whether iPasts expressed key proteins of the astrocyte lineage, such as GFAP and S100B (Figure 4A, left panel, S2C and S3K). Our results showed that while iPasts are predominantly GFAP+ (> 80%), they are almost exclusively S100B+ (>95%) (Figure 4, right panel). Based on this result, we next investigated whether iPasts expressed CD44 (Figure 4B, left panel, Figures S2D and S3I), as astrocytes co-expressing GFAP, S100B and CD44 are considered to be relatively immature astrocytes [46]. GFAP+ iPasts were also positive for CD44, confirming that iPasts are most likely immature astrocytes. This notion was also supported by the expression of Vimentin (Figure 4C left panel, Figures S2E and S3G), which is expressed by immature astrocytes [47]. We next examined the expression of AQP4 (Figure 4C left, Figures S2E and S3G), a water channel which is expressed during astrocytic maturation [15]. However, we found that only a sub-population of astrocytes expressed AQP4 (less than 10%). On the other hand, GJA1, a protein of intercellular gap junction complexes, which is expressed in astrocytes during the perinatal period [48], was strongly expressed (Figure 4D, Figures S2C and S3J), in particular at the junction between two iPasts (Figure 4D-right panel). We also confirmed the expression of SLC1A2 and GRIA1, which regulate glutamate dynamics in astrocytes (Figure 4E,F, Figures S2F,G and S3D,F).

These results demonstrate that iPasts express key markers of astrocytic identity and suggest the functionality of iPasts.

### 3.5. iPasts Can Become Reactive Astrocytes

**Figure 5:**
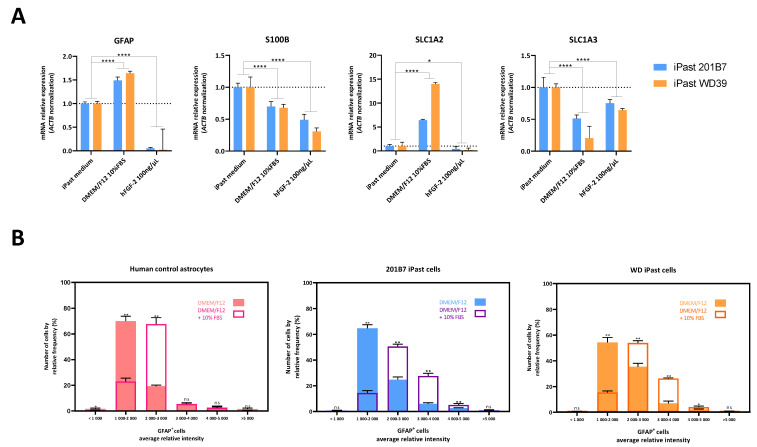
Effect of the Culture Medium on the Transcription of Various Genes in iPasts. (**A**) Cells were cultured in iPast medium, DMEM/F12 with 10% fetal bovine serum (FBS) or 100 ng/mL FGF-2. Quantification of indicated mRNAs was assessed by quantitative RT-PCR. Values were normalized to *ACTB* and represent the fold change ratio value (value/iPSC value). *n* = 3 passages. Statistical test: One-way ANOVA with post-hoc Tukey. **** and * indicate *p*-values (p) < 0.0001 ≤ 0.05, respectively. (**B**) Analysis of the distribution of GFAP+-iPasts depending on the individual mean GFAP intensity, using automatic immunofluorescence analysis by IN Cell Analyzer. Control human astrocytes, 201B7 and WD39 iPasts were cultured in iPast culture medium or DMEM/F12 plus 10% FBS. *n* = 3 passages. Statistical test used: Kolmogorov-Smirnov test. ** and * indicate *p*-values (p) < 0.01 and ≤ 0.05, respectively while ns stands for non significant.

To investigate the capacity of iPasts to turn from quiescent to reactive astrocytes, we questioned the effect of culture medium regarding the transcription of key genes of the astrocytic lineage. Therefore, we compared the influence of our differentiation medium (iPast medium) vs. DMEM/F12 supplemented with 10% FBS or 100 ng/µL FGF-2 on the transcription of *GFAP*, *S100B*, *SLC1A2 and SLC1A3* in iPasts (Figure 5A). When cultured with FGF-2, iPasts showed a reduced transcription of these four genes. Regarding *GFAP*, this result is consistent with previous work showing that FGF-2 decreases astrocyte activation by reducing the level of GFAP expression [49]. Conversely, iPasts cultured with FBS showed an increased transcription of *GFAP* and *SLC1A2*, genes associated with the status of activated mature astrocytes but had fewer transcripts for *S100B* and *SLC1A3* than iPasts grown in our medium. Using automatic image analysis by IN Cell Analyzer, we analyzed the distribution of GFAP+-iPasts depending on the individual mean GFAP intensity. iPasts were cultured in iPast medium or in the presence of 10% FBS and human fetal astrocytes cell culture (FAC) were used as positive control (Figure 5B). iPasts were classified into six classes of GFAP-fluorescence. Our data confirmed that culture in 10% FBS induced a significant shift toward a higher GFAP-immunofluorescence in the three sources of astrocytes examined.

These experiments indicate that iPasts can adapt their transcriptional profile depending on the medium and turn from quiescent to reactive astrocytes if cultured with FBS.

### 3.6. iPasts are Functional

**Figure 6:**
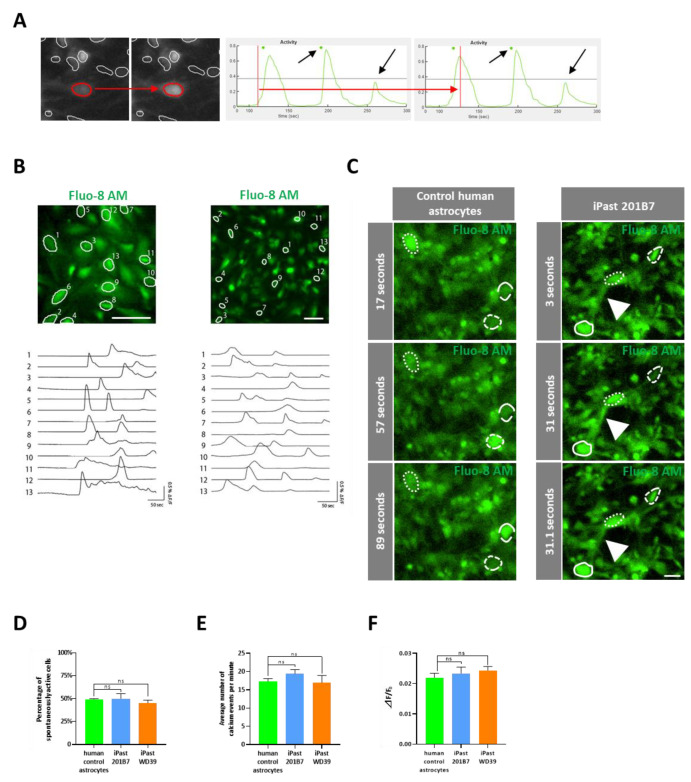
Spontaneous Calcium Fluctuations in Control Human Astrocytes, 201B7 and WD39 iPasts. (**A**) Representative ROI (red line) after automatic preprocessing by miniscope 1-photon-based calcium imaging signal extraction pipeline (MIN1PIPE) software, before and at the peak of fluorescence (left pictures). Illustration of the method of analysis: the determination of a threshold and duration of calcium fluctuation for each same event results in considering 2 calcium transients (green dots) rather than 3 peaks (black arrows) (right panels). (**B**) Representative calcium transients in human control-astrocytes (left) and iPasts (right). Time traces of corresponding cells delineated with white outlines. Scale bars: 20 µm. (**C**) Representative experiments of Fluo-8 AM-stained human control astrocytes and 201B7 iPasts at different time points. The dotted circles indicate calcium transients captured during the acquisition, while white arrowheads point to a transient calcium fluctuation along an intercellular process. Scale bar: 10 µm. (**D**) Percentage of spontaneously active cells in human control-astrocytes, 201B7 iPasts and WD39 iPasts. (**E**) Average number of calcium events per minute in human control-astrocytes, 201B7 iPasts and WD39 iPasts. (**F**) Average variation of calcium transients amplitude for human control-astrocytes, 201B7 iPasts and WD39 iPasts. Data shown are represent mean ± SEM. *n* = 3 passages. Statistical test: Mann-Whitney test. ns stands for non significant.

Based on recent reports which underscored the importance of calcium levels in astrocytes to modulate neuronal functionality [50,51], in order to elucidate the functionality of iPasts, we first investigated astrocytic calcium signaling, indicative of the intercellular communication network between astrocytes [52,53,54]. To monitor calcium dynamics, we performed calcium imaging using Fluo-8 AM, a calcium indicator that has twice the fluorescence intensity of Fluo-4 AM. Image analysis was performed with custom scripts based on MIN1PIPE [28] to facilitate semi-automated detection and analysis of calcium dynamics in astrocyte (Figure 6A). iPasts displayed spontaneous and periodic calcium transients, as observed in control human FA (Figure 6B, Figure S2H and Supplementary movie 1). This result is in favor of an astrocytic phenotype, given that neuronal calcium activity is much faster. The calcium activity could be observed both in the soma and processes of astrocytes (Figure 6C). There were no differences in the percentage of spontaneously active cells (Figure 6D), the number of calcium events per minute (Figure 6E) and the amplitude of calcium events (Figure 6F) between the human control astrocytes and iPasts. These data imply that iPasts exhibit calcium dynamics features similar to primary human fetal astrocytes.

**Figure 7:**
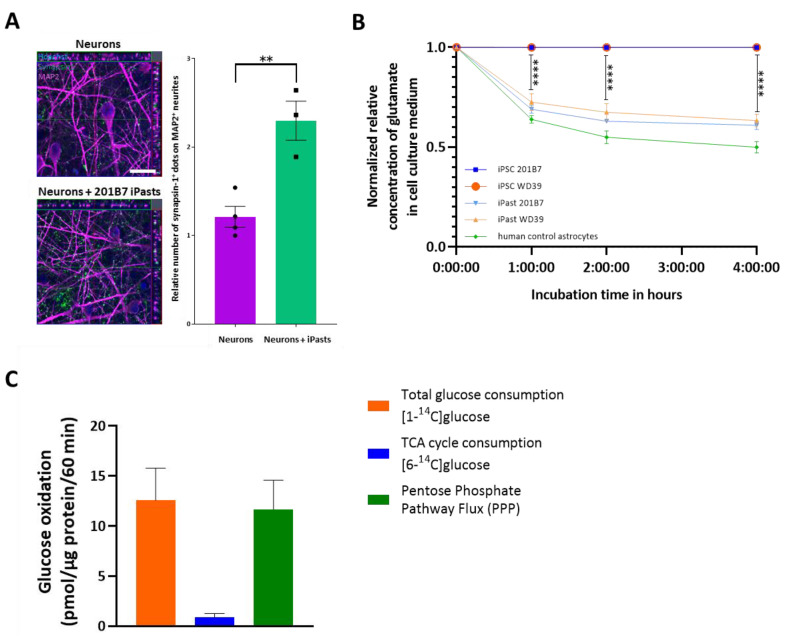
iPasts are Functional Cells. (**A**) Healthy cortical neurons in indirect co-culture with iPasts. Neurons were immunostained for Synapsin-1 (green) and MAP2 (magenta). Histograms show the analysis of Synapsin-1+ dots in MAP2+ neurons. *n* = 3 independent experiments. Scale bar: 20 µm. Individual data are shown as • and ▪ and error bars are mean ± SEM. Statistical test used: Student’s *t*-test. ** indicates *p*-value (p) < 0.01. (**B**) Glutamate uptake of iPast lines was compared to iPSCs (negative control) and human control astrocytes (positive control) at 1, 2 and 4 h after adding glutamate at 250 µM. *n* = 9 passages. Statistical test used: Mann-Whitney test. **** indicates *p*-value (p) < 0.0001. (**C**) Rates of ^14^CO_2_ production from the oxidation of [1-^14^C]-glucose and [6-^14^C]-glucose and rate of the PPP in iPasts. Values are means ± SEM for quadruplicate flasks.

We next addressed the effect of iPasts on neurons. Indirect co-culture of iPasts with iPSC-derived neurons revealed that Synapsin1 expression in neurons was drastically upregulated in the presence of iPasts (Figure 7A and Figure S3E,G). Given that Synapsin1 has an essential function in neuronal synapse regulation [55,56,57,58], our results indicate that iPasts have a supporting effect on neuronal maturation.

In connection with the high expression in iPasts of SLC1A2 glutamate transporter (Figure 4E), which contributes to ~90% of glutamate uptake by astrocytes [45] and because glutamate clearance from the synaptic cleft terminates glutamatergic transmission in vivo and prevents glutamatergic excitotoxicity, we next assessed whether iPasts could uptake glutamate. Using the starting iPSC lines as negative controls and the human astrocyte line as a positive control, we exposed iPasts to 250 µM of glutamate and quantified the glutamate uptake one, two and four hours after the incorporation of glutamate (Figure 7B). Our experiments demonstrated that iPasts were capable of taking up glutamate in a time-dependent manner and in the same order of magnitude that the human fetal control astrocytes, in contrast with the starting iPSC lines.

Astrocytes have a characteristic glucose metabolism in which glycolysis dominates tricarboxylic acid (TCA) cycle activity. Accordingly, pyruvate is mainly converted into lactate, which is transported from astrocytes to neurons, through monocarboxylate transporters (MCTs). In neurons, lactate is subsequently oxidized into pyruvate by LDH1, resulting in a neuronal TCA cycle activity that predominates over the intracytoplasmic glycolysis. Therefore, we finally questioned whether iPasts could have any glycolytic activity. For this purpose, we quantified the rate of [1-^14^C]-glucose and [6-^14^C]-glucose oxidation. [1-^14^C]-glucose is metabolized both in TCA cycle and pentose-phosphate pathway (PPP), whereas [6-^14^C]-glucose is metabolized only in TCA cycle. iPasts exhibited a substantial metabolic capacity of total glucose oxidation but a negligible oxidative metabolism through the TCA cycle, indicative of a low mitochondrial glycolytic activity (Figure 7C). These results in iPasts mirror the metabolic preferences observed in rodent astrocytic cells in vitro [59,60].

## 4. Discussion

In the present study, we characterized iPasts obtained from iPSC lines by our original protocol and provided pieces of evidence of essential astrocytic functions in iPasts. The transcriptomic and protein expression analyses suggest that iPasts are immature astrocytes. Nonetheless, we found that iPasts exhibited several important aspects of functional astrocytes, including calcium dynamics, neuronal synapse maturation, glutamate uptake activity and glucose metabolism.

During the embryonic development, astrogenesis follows neurogenesis [61]. The transition from neurogenesis to astrogenesis is a complex process governed by genetic/epigenetic intracellular mechanisms [61,62,63,64,65,66] and by extracellular factors [63,67]. In our protocol, although we did not intentionally modulate any signaling involved in astrogenesis, the long-term culture of iPSC-derived neural cells appears to mirror the developmental transition from neurogenesis to astrogenesis. The choice of our initial culture method, that is, adherent culture vs. embryoid bodies, is based on the relevant literature. First, the expression of *GFAP* was lower in astrocytes generated from adherent cultures compared with astrocytes generated from embryoid bodies [15,68]. Accordingly, a large proportion of iPasts express GFAP. Second, adherent culture seemed less relevant to the natural developmental process characterized by numerous intercellular contacts than 3D cultures such as embryoid bodies and neurospheres. Finally, it should be emphasized that the embryoid bodies approach allowed us to compare our RNA-Seq data set with the results of other research groups that also used non-adherent cultures.

Our protocol has the advantage of generating a highly homogeneous population of iPasts, which would be helpful for disease modeling in vitro (Figure S1B,C lower panels). In our present protocol, the three final passages are likely to play a crucial role in obtaining high-purity cultures of astrocytes by eliminating neuronal cells. Indeed, the neuronal marker TUBB3 was significantly downregulated from A0-step to A4-step (Figure S1C). Thus, this final-passages stage constitutes an improvement of the present method compared with other protocols [16,18] for the enrichment of iPSCs-derived astrocytes. On the other hand, this purity might also interfere with the functionality of astrocytes. Previous studies have indeed reported the important role of neurons in astrocyte differentiation and gene expression [69,70,71]. In vitro, neurons can modulate the expression of glutamate transporters in astrocytes. Polygonal cortical astrocytes turn into SLC1A2+-astrocytes with a stellar shape when co-cultured with neurons [69]. In addition, previous reports showed that the expression of SLC1A2 and SLC1A3 in astrocytes was modulated by neuronal soluble factors [72,73], indicating that normal protein expression by astrocytes requires neuronal upstream signaling. In our cultures, the expression of some markers, including *ALDOC*, *ALDH1L1* and *GRM5,* were reduced in parallel with the progressive elimination of neuronal cells. This may suggest that iPasts co-cultured with neurons could become transcriptionally more mature. While we have demonstrated the effect of co-culture on neuronal maturation, its effect on astrocytic maturation should be investigated in a future study.

When considering the robustness of our iPast protocol, our gene expression analyses revealed several differences between 201B7 and WD39 iPSC lines-derived iPasts in terms of timing (e.g., A0 vs. a later passage) or magnitude of the changes observed (see e.g., *AQP4*, *NFIA* or *SLC1A3)*. It has already been reported that 201B7 and WD39 iPSC lines differ in their propensity to naturally differentiate into different lineages. Indeed, while 201B7 iPSCs can spontaneously differentiate easily toward the ectodermal lineage, WD39 iPSCs preferentially express markers characteristic of the mesendodermal lineage [74]. Thus, it is possible that the terminal differentiation of iPSCs into iPasts is partly governed by the clonal variations of iPSC lines. Nevertheless, the treatment with dual SMAD inhibitors along with GSK3β inhibitor can overcome these clonal variations to some extent [38] and the whole gene expression pattern of iPasts from each iPSC lines are very similar (Figure 2D). Thus, despite some differences, our iPast protocol can generate homogenous astrocytes, independently from the starting iPSC clones and should be helpful for generating astrocytes from disease-specific iPSC lines.

Regarding glycolytic metabolism, we provided pieces of evidence of a highly glycolytic metabolism but a low mitochondrial activity in iPasts. We can hypothesize that this low mitochondrial metabolism mirrors the in vivo actual metabolism of glucose. In fact, neurons do not have any direct contacts with micro-vessels despite their strict dependence on a continuous supply of glucose and oxygen through the cerebral blood flow. In contrast, 99% of the surfaces of brain capillaries are covered by astrocytic processes (end-feet), indicating that all essential nutrients supplied from the cerebral circulation must interact with astroglia before reaching the neurons [75]. As astrocytes are interposed between neurons and cerebral micro-vessels, we can assume that glycolytic metabolism, among other metabolic functions, occurs in astrocytes for the benefit of neurons [60]. Considering that most evidence is based on in vitro studies using rodent neural cell cultures that might not be an appropriate model for human brain cells [76], the high glycolytic metabolic activity associated with lactate production might be a characteristic of rodent cell cultures only. Importantly, however, the present study demonstrated for the first time that human astrocytes may also possess a high glycolytic activity.

The use of differentiated cells from iPSCs technology, as in the present report, enables the evaluation of human astroglia in vitro *and* their relationship with neurons, through co-cultures.

## 5. Conclusions

In the present study, we report a culture protocol to generate astrocytes from human iPSCs (iPasts). Based on the expression of various markers, iPasts are likely to correspond to immature astrocytes. However, iPasts showed functional characteristics of astrocytes such as spontaneous calcium oscillation, glutamate uptake, dominant anaerobic glycolytic activity and supporting effects on neuronal maturation. Thus, the availability of iPasts will help to address the contribution of astrocytes to the development of neurodegenerative diseases and open the possibility of testing candidate drugs in an in vitro context firstly, before eventually developing in vivo models.

## Supplementary Materials

The following are available online at https://www.mdpi.com/2073-4409/9/12/2680/s1, Figure S1: Quality control of the lineage of the cell lines by immunofluorescence, Figure S2: Characterization of the iPast lines differentiated from WD39 iPSC line, Figure S3: Controls for immunofluorescent stainings, Table S1: Published RNA-seq data used in this study, Table S2: Gene sets used in Figure 2E, Table S3: List of primers and antibodies used, Video S1: The movie shows representative calcium imaging data from human control-astrocytes and iPasts.
