# Supplementary material for: Human Astrocytes Model Derived from Induced Pluripotent Stem Cells
Source: Cells. 2020 Dec 13;9(12):2680. doi: 10.3390/cells9122680 (PMC7763297; doi:10.3390/cells9122680)
Supplement: Supplementary file 1 [file cells-09-02680-s001.zip › Supplementary resubmit-2/Supplementary legends proofread.docx]

**Supplementary Figure 1:** Quality control of the lineage of the cell lines by immunofluorescence. **A.** Control of the stemness of 201B7 (left panel) and WD39 (right panel) iPSC lines for indicated markers. Single immunostaining for Tra1-81 (red), double staining for NANOG (green) and SSEA4 (red), or for SOX2 (green) and OCT4 (red) are shown. Nuclei were stained with Hoechst 33258. Scale bars: 100 µm. **B.** Oligodendrocytic, neuronal and microglial induction is very limited in the 201B7 iPast line. Cells were stained for GFAP (red), O4 (magenta) and TUBB3 (left panel-green) or OLIG2 (central panel-green). Right panel shows a representative staining for AIF1 protein. Nuclei were stained with Hoechst 33258. n = 3 passages. Scale bars: 150 µm. The lower histograms present the quantitative RT-PCR analysis for TUBB3 (lower left) and OLIG2 (lower right). Values represent fold change (value/iPSCs) normalized to ACTB. n = 3 passages. HB stands for human brain. **C.** Control of the low oligodendrocytic, neuronal and microglial induction in WD39 iPast line. Cells were stained for GFAP (red), O4 (magenta) and TUBB3 (left panel-green) or OLIG2 (central panel-green). Right panel shows a representative staining for AIF1 protein. Nuclei were stained with Hoechst 33258. n = 3 passages. Scale bars: 150 µm. Lower histograms show the quantitative RTPCR analysis for TUBB3 (lower left panel) and OLIG2 (lower right panel). Values represent fold change (value/iPSC) normalized to ACTB. n = 3 passages. HB stands for human brain.

**Supplementary Figure 2:** Characterization of the iPast lines differentiated from WD39 iPSC line. **A.** Quantitative RT-PCR of indicated mRNA for the WD39 iPSC line. FA stands for control fetal astrocytes and HB for human brain. Values are normalized to ACTB quantification and represent the fold change ratio value/iPSC value. n = 3 passages in duplicate. Data shown are mean ± SEM. Statistical test used: Wilcoxon Matched-pairs test. **B.** Heatmap recapitulating the gene transcription analyses by quantitative RT-PCR shown in A. iPast A4 status is highlighted by the green box. Values are logarithm to the base 2 of fold change from A. **C.** Representative by immunofluorescent stainings for the WD39 iPasts for GFAP (red), GJA1 (green), and S100B (magenta). Scale bar: 20 µm. **D.** Representative immunofluorescent stainings in WD39 iPasts for GFAP (red) and CD44 (green). Scale bar: 20 µm. **E.** Representative immunofluorescent stainings in WD39 iPasts for VIMENTIN (VIM) (red) and AQP4 (green). Scale bar: 20 µm. **F.** Representative immunofluorescent stainings in WD39 iPasts for GFAP (red) and SLC1A2 (green). Scale bar: 20 µm. **G.** Representative immunofluorescent stainings in WD39 iPasts for GFAP (red) and GRIA1 (green). Scale bar: 20 µm. Nuclei are stained with Hoechst 33258. n = 3 independent astrocytes induction experiments. **H.** Representative experiment of calcium transients detected in WD39 iPasts by Fluo-8 AM at 9, 30, 150 and 195 seconds of imaging.

**Supplementary Figure 3:** Controls for immunofluorescent stainings. **A.** Brain section from a 2-month-old Swiss mouse stained for GFAP (red) and OLIG2 (green). Scale bar: 100 µm. **B.** Colony of 201B7 iPSCs stained for O4 (red). Scale bar: 50 µm. **C.** iPSC-derived microglial cells co-cultured with neurons were stained for anti-mouse TUBB3 (red) and AIF1 (green). Scale bar: 100 µm. **D.** Brain section from a 2-month-old Swiss mouse stained for SLC1A2 (red). Scale bar: 100 µm. **E.** Brain section from a 2-month-old Swiss mouse stained for MAP2 (red) and anti-rabbit TUBB3 (green). Scale bar: 100 µm. **F.** Brain section from a 2-month-old Swiss mouse stained for GRIA1 (red). Scale bar: 100 µm. **G.** Brain section from a 2-month-old Swiss mouse stained for SYNAPSIN-1 (red) and AQP4 (green). Scale bar: 100 µm. **H.** Colony of 201B7 iPSCs stained for VIMENTIN (red). Scale bar: 50 µm. **I.** Colony of 201B7 iPSCs stained for CD44 (red) and SOX10 (green). Scale bar: 30 µm **J.** Colony of 201B7 iPSCs stained for GJA1 (green). Scale bar: 100 µm. **K.** Brain section from a 2-month-old Swiss mouse stained for S100B (green). Scale bar: 50 µm.

**Supplementary Movie 1:** The movie shows representative calcium imaging data from human control-astrocytes and iPasts.
