# Supplementary material for: Human Astrocytes Model Derived from Induced Pluripotent Stem Cells
Source: Cells. 2020 Dec 13;9(12):2680. doi: 10.3390/cells9122680 (PMC7763297; doi:10.3390/cells9122680)
Supplement: Supplementary file 1 [file cells-09-02680-s001.zip › Supplementary resubmit-2/Supplementary Table 1.docx]

Supplementary Table 1: Published RNA-seq data used in this study

| Report | SRA ID | Description |
| --- | --- | --- |
| TCW *et al.* | SRR5454004 | 3651_Astros |
|  | SRR5454006 | 9319_Astros |
|  | SRR5454007 | 9429_Astros |
|  | SRR5454009 | BJ_Astros |
| Tchieu *et al.* | SRR8062766-7 | NFIA-Astro-1 |
|  | SRR8062768-9 | NFIA-Astro-2 |
|  | SRR8062770-1 | NFIA-Astro-3 |
|  | SRR8062772-3 | NFIA-Astro-4 |
|  | SRR8062774-5 | NFIA-Astro-5 |
|  | SRR8062776-7 | NFIA-Astro-6 |
|  | SRR8062778-9 | NFIA-Astro-7 |
|  | SRR8062780-1 | NFIA-Astro-8 |
|  | SRR8062782-3 | NFIA-Astro-9 |
|  | SRR8062784-5 | NFIA-Astro-10 |
|  | SRR8062786-7 | NFIA-SC-Astro-1 |
|  | SRR8062788-9 | NFIA-SC-Astro-2 |
|  | SRR8062790-1 | NFIA-SC-Astro-3 |
| Zhang *et al.* | SRR2557083 | Fetal ctx 1 astro |
|  | SRR2557084 | Fetal ctx 2 astro |
|  | SRR2557085 | Fetal ctx 3 astro |
|  | SRR2557086 | Fetal ctx 4 astro |
|  | SRR2557087 | Fetal ctx 5 astro |
|  | SRR2557088 | Fetal ctx 6 astro |
|  | SRR2557089 | 8yo ctx astro |
|  | SRR2557090 | 13yo ctx astro |
|  | SRR2557091 | 16yo ctx astro |
|  | SRR2557092 | 21yo ctx astro |
|  | SRR2557093 | 22yo ctx astro |
|  | SRR2557094 | 35yo ctx astro |
|  | SRR2557095 | 47yo ctx astro |
|  | SRR2557096 | 51yo ctx astro |
|  | SRR2557097 | 53yo ctx astro |
|  | SRR2557098 | 60yo ctx astro |
|  | SRR2557099 | 63yo ctx 1 astro |
|  | SRR2557100 | 63yo ctx 2 astro |
