# Supplementary material for: Human Astrocytes Model Derived from Induced Pluripotent Stem Cells
Source: Cells. 2020 Dec 13;9(12):2680. doi: 10.3390/cells9122680 (PMC7763297; doi:10.3390/cells9122680)
Supplement: Supplementary file 1 [file cells-09-02680-s001.zip › Supplementary resubmit-2/Supplementary Table 2.docx]

| List of the 239 genes associated with astrocyte identity in iPSC-derived astrocytes and human primary astrocytes retrieved from Tchieu *et al.* Table S1. | | | | |
| --- | --- | --- | --- | --- |
| *GSTM1*  *SLC7A10*  *CMTM5*  *APOE*  *MFGE8*  *SLC25A18*  *SDC4*  *FGFR3*  *ALDOC*  *RAB34*  *GPR37L1*  *NTSR2*  *AGT*  *FKBP10*  *SLC13A5*  *PYGM*  *SLC6A11*  *HES5*  *ALDH1L1*  *HHATL*  *SYPL2*  *PRODH*  *LRIG1*  *TST*  *RLBP1*  *MLC1*  *ACSS1*  *SMPD2*  *MMD2*  *DBI*  *TUBB2B*  *SLC9A3R1*  *HEPACAM*  *SELENBP1*  *S100A1*  *TTYH1*  *GJA1*  *GJB6*  *GFAP*  *ATP1A2*  *PSAT1*  *ENTPD2*  *PYGB*  *GPAM*  *PLEKHB1*  *PHGDH*  *ADORA2B*  *GLI3* | *EZR*  *CHI3L1*  *ATP1B2*  *SH3PXD2B*  *BCAN*  *GSTM5*  *PHKG1*  *GLI2*  *FADS2*  *VEGFA*  *HTRA1*  *PSD2*  *RNF182*  *MASP1*  *SFXN5*  *EFHD1*  *GPRC5B*  *ACOT1*  *HEPH*  *SLC1A3*  *SREBF1*  *SLC15A2*  *PDK4*  *GRIN2C*  *LCAT*  *PPP1R3C*  *CTH*  *SOX9*  *GPC5*  *SLC39A12*  *IGFBP2*  *AASS*  *NWD1*  *GLUD1*  *PLCD4*  *S100B*  *PLD2*  *PRDM16*  *PRDX6*  *ASRGL1*  *ABHD3*  *PAQR8*  *SLC27A1*  *PADI2*  *BMPR1B*  *DAAM2*  *SLC1A2*  *OPLAH* | *GAS1*  *EDNRB*  *LGR4*  *MGST1*  *ATP13A4*  *EPHX2*  *SLC4A4*  *IL33*  *CSPG5*  *SOX21*  *EFS*  *GLDC*  *ACSBG1*  *FGFR1*  *SLC14A1*  *ADHFE1*  *SLC41A1*  *ELOVL2*  *EGFR*  *FBXO2*  *TSC22D4*  *FGF1*  *NTRK2*  *PAX6*  *SLC7A11*  *KLF15*  *DIO2*  *PHKA1*  *ID4*  *CBS*  *RPE65*  *GDF10*  *POLR3H*  *ACOT11*  *MSI1*  *ABTB2*  *RGS20*  *RHOBTB3*  *NKX2-2*  *FABP7*  *RFX4*  *MEIS1*  *MRAS*  *GLIS3*  *MAOB*  *FADS1*  *SOX2*  *NPAS3* | *PDLIM4*  *KCNJ16*  *FZD2*  *ABCD2*  *DTNA*  *EYA1*  *GABRG1*  *DLG5*  *DMRTA2*  *VAV3*  *TTPA*  *NAT1*  *EMX2*  *DDAH1*  *ACSL6*  *ACSS2*  *EPS8*  *WNT7A*  *FZD1*  *CYP7B1*  *FABP5*  *APPL2*  *RORB*  *FARS2*  *PRKD1*  *AGPAT5*  *SLC13A3*  *LSAMP*  *AGL*  *AQP4*  *PDPN*  *CTSO*  *CHPT1*  *PGM2*  *ITIH3*  *MYO6*  *HOPX*  *EPS15*  *NR2E1*  *CROT*  *HSD11B1*  *RAB30*  *CLMN*  *LGI4*  *CBR3*  *LHX2*  *ADK*  *PM20D1* | *UGP2*  *ACSL3*  *SPIRE1*  *CXCL14*  *GNAO1*  *TIMP4*  *PRRX1*  *CHST2*  *PPIL6*  *TPRKB*  *SMPDL3A*  *SPAG5*  *MTMR11*  *HAPLN1*  *GPLD1*  *KCTD14*  *VCAM1*  *PFN4*  *TOM1L1*  *SPAG1*  *CDH19*  *ACAD11*  *AOX1*  *IL18*  *KCNN2*  *DMP1*  *LXN*  *PAPSS2*  *GHR*  *NAT8*  *PLA2G7*  *AQP9*  *GAPDH*  *ACTB*  *HPRT1*  *TBP*  *C1orf43*  *CHMP2A*  *EMC7*  *GPI*  *PSMB2*  *PSMB4*  *RAB7A*  *REEP5*  *SNRPD3*  *VCP*  *VPS29* |

Supplementary Table 2 : Gene set use in Figure 2E
