# Supplementary material for: Human Astrocytes Model Derived from Induced Pluripotent Stem Cells
Source: Cells. 2020 Dec 13;9(12):2680. doi: 10.3390/cells9122680 (PMC7763297; doi:10.3390/cells9122680)
Supplement: Supplementary file 1 [file cells-09-02680-s001.zip › Supplementary resubmit-2/Supplementary Table 3 proofread.docx]

Supplementary Table 3: List of primers and antibodies used

| *List of primers used in alphabetical order* | | |
| --- | --- | --- |
| **Primers** | **Forward** | **Reverse** |
| ACTB | TGAAGTGTGACGTGGACATC | GGAGGAGCAATGATCTTGAT |
| ALDOC | GCCAAATTGGGGTGGAAAACA | TTCACACGGTCATCAGCACTG |
| ALDH1L1 | GCTCCATCATCTATCACCCGT | ATCTCCGTGAATGAGGGTCCA |
| AQP4 | TGCCAGCTGTGATTCCAAACGG | TGGGCCCAACCCAATATATCCAA |
| CD44 | CTCCAGTGAAAGGAGCAGCA | AGCAGGGATTCTGTCTGTGC |
| GFAP | TGTGAGGCAGAAGCTCCAGGATGA | AGGGTGGCTTCATCTGCTTCCTGT |
| GJA1 | GGTGACTGGAGCGCCTTAG | GCGCACATGAGAGATTGGGA |
| GRM3 | CAGCGCCAAACTCAGTGATA | GGGCTTCTGCAACAGTTCTC |
| GRM5 | AGACCAACCGTATTGCAAGG | TGAACGCATAGAAGGTGCAG |
| NFIA | AGCTCATGGAGCGGCAATAG | ATTCATCCTGGGTGAGACAGAG |
| OLIG2 | GTTCTCCCCTGAGGCTTTTC | AGAAAAAGGTCATCGGGCTC |
| S100B | GTGGCCCTCATCGACGTTTT | ACCTCCTGCTCTTTGATTTCCTCT |
| SLC1A2 | AGGCGCTAAAGGGCTTACC | GGGCATATTGTTGGCACCTTC |
| SLC1A3 | CCGCTGTCATTGTGGGTACA | CCTCATCAGAAGTTCCCCAGG |
| SLC6A13 | GGATGGATAGCAGGGTCTCA | AACCTCCAGACGTTGCCTAA |
| SOX2 | GGGAAATGGGAGGGGTGCAAAAG | TTGCGTGAGTGTGGATGGGATTG |
| SOX9 | AGGAAGTCGGTGAAGAACGG | AAGTCGATAGGGGGCTGTCT |
| TUBB3 | ATTTCATCTTTGGTCAGAGTGGGGC | TGCAGGCAGTCGCAGTTTTCAC |

| *List of antibodies used in alphabetical order* | | |
| --- | --- | --- |
| **Antibodies** | **Dilution** | **RRID** |
| Rabbit anti-AIF1 | 1 : 600 | Wako Cat# 019-19741, RRID:AB_839504 |
| Rabbit anti-AQP4 | 1 : 300 | Santa Cruz Biotechnology Cat# sc-20812, RRID:AB_2274338 |
| Mouse anti-CD44 | 1 : 1,000 | Abcam Cat# ab6124, RRID:AB_305297 |
| Rat anti-GFAP | 1 : 700 | Thermo Fisher Scientific Cat# 13-0300, RRID:AB_2532994 |
| Rabbit anti-GJA1 | 1 : 200 | Sigma-Aldrich Cat# C6219, RRID:AB_476857 |
| Mouse anti-GRIA1 | 1 : 100 | Millipore Cat# MAB2263, RRID:AB_11212678 |
| Chicken anti-MAP2 | 1 : 100 | Thermo Fisher Scientific Cat# PA1-10005, RRID:AB_1076848 |
| Rabbit anti-NANOG | 1 : 250 | Cosmo Bio Cat# REC-RCAB004P-F, RRID:AB_2714012 |
| Mouse anti-O4 | 1 : 25 | Millipore Cat# MAB345, RRID:AB_11213138 |
| Mouse anti-OCT4 | 1 : 500 | Santa Cruz Biotechnology Cat# sc-5279, RRID:AB_628051 |
| Rabbit Anti-OLIG2 | 1 : 500 | Millipore Cat# AB9610, RRID:AB_570666 |
| Mouse anti-S100B | 1 : 500 | Sigma-Aldrich Cat# S2532, RRID:AB_477499 |
| Rabbit anti-SLC1A2 | 1 : 100 | Abcam Cat# ab41621, RRID:AB_941782 |
| Rabbit anti-SOX2 | 1 : 200 | Abcam Cat# ab59776, RRID:AB_945584 |
| Mouse anti-SSEA4 | 1 : 250 | Abcam Cat# ab16287, RRID:AB_778073 |
| Mouse anti-SYNAPSIN-1a/b | 1 : 100 | Santa Cruz Biotechnology Cat# sc-398849, RRID:AB_2744495 |
| Mouse anti-TRA-1-81 | 1 : 500 | Millipore Cat# MAB4381, RRID:AB_177638 |
| Rabbit Anti-TUBB3 | 1 : 200 | Abcam Cat# ab18207, RRID:AB_444319 |
| Rat anti-VIMENTIN | 1 : 200 | R and D Systems Cat# MAB2105, RRID:AB_2241653 |
| Alexa Fluor 555 Goat anti-Chicken IgY | 1 : 500 | Thermo Fisher Scientific Cat# A-21437, RRID:AB_2535858 |
| Alexa Fluor 488 Donkey Anti-Mouse IgG | 1 : 500 | Molecular Probes Cat# A-21202, RRID:AB_141607 |
| Alexa Fluor 488 Donkey Anti-Rabbit IgG | 1 : 500 | Thermo Fisher Scientific Cat# A-21206, RRID:AB_2535792 |
| Alexa Fluor 555 Donkey anti-Mouse IgG | 1 : 500 | Thermo Fisher Scientific Cat# A-31570, RRID:AB_2536180 |
| Alexa Fluor 594 Donkey Anti-Rabbit IgG | 1 : 500 | Molecular Probes Cat# A-21207, RRID:AB_141637 |
| Alexa Fluor 594 Donkey anti-Rat IgG | 1 : 500 | Thermo Fisher Scientific Cat# A-21209, RRID:AB_2535795 |
| Alexa Fluor 647 Donkey anti-Mouse IgG | 1 : 500 | Thermo Fisher Scientific Cat# A-31571, RRID:AB_162542 |
